# The DiReCT study - improving recruitment into clinical trials: a mixed methods study investigating the ethical acceptability, feasibility and recruitment yield of the cohort multiple randomised controlled trials design

**DOI:** 10.1186/1745-6215-15-398

**Published:** 2014-10-16

**Authors:** David A Richards, Sarah Ross, Sarah Robens, Gunilla Borglin

**Affiliations:** University of Exeter Medical School, Haighton Building, St Luke’s Campus, Heavitree Road, Exeter, EX1 2 LU UK; Research and Development Department, Devon Partnership NHS Trust, Wonford House Hospital, Dryden Road, Exeter, EX2 5AF UK; Department of Care Science, Malmo University, SE 205 06 Malmö, Sweden

## Abstract

**Background:**

The ‘cohort multiple Randomised Controlled Trial’ (cmRCT) design has been proposed as a potential solution to poor recruitment into clinical trials. The design randomly selects participants eligible for experimental treatments from a pre-enrolled cohort of patients, recruiting participants to multiple trials from a single cohort. Controls remain unaware of their participation in specific trials.

**Methods:**

We undertook a mixed methods study to determine the ethical acceptability, the proportion of patients in a routine service consenting to cohort participation, the proportion of these who would consent to being hypothetically randomly selected to receive new treatments, and the views of clinicians on the acceptability of the design. We submitted our cmRCT design for ethical review and recruited participants from people with anxiety and depression attending a community mental health service of twenty-one clinicians. We recorded the proportion of patients who were offered participation in the DiReCT study and the proportion that consented to researcher contact, medical record sharing, and who accepted to be randomly allocated to active treatment procedures in future hypothetical unspecified clinical trials. We used a thematic framework analysis to analyse clinician interviews.

**Results:**

We obtained a favourable ethical opinion from the UK Health Research Authority. Clinicians approached 131/752 (17%) potentially eligible participants for consent. Of these 131, 84 (64%) initially consented to be contacted by a researcher and all but one consented to being randomised into future trials. We confirmed consent for 71 (54%) of participants approached by clinicians, of whom 69 (53%) consented to being randomised into hypothetical future trials, 9% (69/752) of all potentially eligible patients. The interviewed clinicians described issues impacting on their ability to recruit participants in terms of clinical concerns for patient wellbeing, work pressure, their views of both general research and the specific DiReCT study, and how they viewed patients’ responses to being offered participation in the study.

**Conclusions:**

The cmRCT system offers the potential to improve the recruitment into clinical trials and is acceptable ethically and to many patients. Overcoming the multiple factors driving the difficulties clinicians experience in patient recruitment is likely to require the application of significant implementation science-informed effort.

## Background

The randomised controlled trial (RCT) is regarded by scientists and health commentators alike as the ‘gold standard’ example [1] of a ‘fair test’ [2] for establishing the effectiveness of a treatment. Nonetheless, recruitment issues, sampling bias, ethics, patient preferences and treatment comparisons can limit the application of the RCT design in practice [3]. For example, many trials struggle to recruit participants. No more than 50% of trials [4–9] recruit to target or on time, with the consequence that some trials may be underpowered, requiring further trials and delaying the time when decisions on whether or not to adopt new interventions can be made. Recruitment to multiple trials usually requires repetitive and wasteful investment in participant identification systems [10]. Patients might decline participation in trials for fear of receiving a treatment they perceive as less desirable than the alternative or because they reject the idea of their treatment being allocated by chance [1, 11].

In the UK, embedding research in clinical services is a core function of the National Health Service (NHS). UK government policy states that every eligible patient attending an NHS treatment programme should be offered the opportunity to participate in clinical research [12]. Governmental purchasers of health care need to demonstrate that they have in place systems and processes to promote the recruitment of patients into research [13]. The National Institute for Health Research (NIHR) in the UK also recognises the importance of increasing opportunities for patients and the public to participate in research to help the NHS become an internationally recognised centre of research excellence [14, 15].

Embedding research into routine clinical practice is one potential method to improve the recruitment of participants into clinical trials. Recruitment methods tailored to routine health delivery systems could avoid repetitive and expensive procedures, provide efficiencies of scale and may overcome poor recruitment yields. The Zelen design [16] was originally proposed as a way of maximising recruitment into clinical trials by eliminating the need for clinicians to have pre-consent conversations with their patients about clinical uncertainty, randomisation and all treatment options in a trial. In this design, participants for a trial are identified and randomised without their knowledge or consent for trial participation. Consent for subsequent treatment allocation is sought after the randomisation procedure has been undertaken. However, despite this method potentially increasing recruitment, the Zelen design has been argued to be unethical since participants have not been given the opportunity to consent to be involved in a clinical trial before one important component of that trial has been undertaken – that is, the random allocation of treatment [17]. Exercising informed choice as to whether one should be randomly allocated to receive a treatment or procedure in a clinical trial is a fundamental principle of ethical research conduct [18], and one might reasonably argue that this should also be applied to those participants contributing data having been randomised or selected to be part of a control group, not just those allocated to the experimental intervention.

Consequently, a number of modifications to the Zelen design [16] have been proposed, including the ‘single consent design’ [17]. In this design, consent for trial participation is only sought from participants who have previously consented to be involved in an observational study. Subsequently, some participants in the observational study are then randomised into an experimental treatment group and specific consent for their participation in the experimental treatment is sought. No further consent is sought from participants randomised to the control condition, who remain unaware of the trial although their data is used to compare the performance of the experimental treatment against the control. As an extension of this, Relton and colleagues [3] have proposed the ‘cohort multiple randomised controlled trial’ (cmRCT) design in which multiple trials can be run from the same cohort using a similar consent procedure as above, but applied to sub-samples of cohort participants carefully selected according to different trial inclusion criteria. Neither of these designs seek *a priori* consent to be part of an experimental evaluation from participants randomised to the treatment as usual conditions and neither design provides information about the trial treatment to people receiving and providing data on treatment as usual in the trial. The UK Medical Research Council has recommended considering such ‘non-standard’ randomised consent designs as a method to overcome challenges for evaluating complex interventions, especially if there is a risk of patient preference bias [19].

In the cmRCT design, patients with the condition of interest are invited to participate in an observational cohort and have their routine outcome data collected by researchers at regular intervals for health research purposes. Those patients who consent to being in the cohort essentially become a pool of ‘research-ready’ potential participants for recruiting directly into RCTs. A key feature of the cmRCT design is how consent is sought for the randomization process. Eligible participants are identified from the observational cohort, and then randomised to either the treatment or control group without prior consent. Consent is only sought from eligible participants who are selected for the experimental treatment arm of the trial on whether they wish to accept the trial treatment or not. The control group consists of cohort participants who were eligible for the experimental treatment if they had been randomly selected, but these people are not told that they could have been randomly selected to receive the experimental intervention, nor that their data is being used for comparative purposes about the trial. They continue to receive treatment as usual, being assessed as part of the longitudinal cohort to provide comparative data. This approach has been described as random selection of ‘some’ of the cohort rather than random allocation of ‘all’ of the cohort [3]. The process of obtaining patient consent from only those selected for the intervention (the ‘offer’ group) aims to replicate the process in real world routine health care where clinicians usually only provide patients with information about their treatment at a time they need the treatment and when the treatment is available.

The cmRCT method has yet to be tested fully in terms of ethical, clinical and patient acceptability, and recruitment efficiency. Relton and colleagues have applied the cmRCT design with a cohort of women in the menopause which received ethics permission [20]. Kwakkenbos and colleagues have recently published a protocol to test the cmRCT method with a rare disease cohort but no results are as yet available [21]. However, neither of these studies have tested the acceptability to patients of their ‘potential for randomisation’ – that is, whether patients would consent to join a cohort on the understanding that they may be randomly selected to receive experimental treatments in currently unspecified clinical trials sometime in the future. As such, therefore, we do not know the acceptability to patients of the cmRCT design, or the likely recruitment yield of implementing such a system.

In Relton and colleagues original proposal [3], they suggested that the cmRCT design be established within an observational cohort. In our DiReCT – improving recruitment to clinical trials - study not only did we aim to test the recruitment yield of the cmRCT design but also the potential for the cmRCT design to contribute to the embedding of research into routine practice. We chose a high-volume mental health service as a case study to determine if patients within a mental health setting would give consent to be part of a cohort when approached by clinicians. Not only did we want to assess the feasibility of a system where patients agree to join a cmRCT cohort within which there is the potential to be selected to receive treatments being tested in RCTs, we also wanted to assess the extent to which mental health clinicians could, or would, undertake cmRCT participant recruitment.

In our hypothetical cmRCT system, specific treatments would not be specified at the time of consent to participation and only those randomly selected to receive experimental treatments would be approached by investigators with details of the specific trial. Control patients would, therefore, provide data to the trial(s) but would not be specifically approached again regarding trial participation. Participants would always be asked for consent to try an intervention if they were selected, but those in the control group, who were not selected, would not be given any information about the trial.

Although there were no actual trials running at the time of the DiReCT study, we wanted to undertake a proof of principle study before potentially investing in setting up the cmRCT cohort. Our study was, therefore, hypothetical in nature in that we were testing the likelihood of participants consenting to our procedures. We wished to undertake this feasibility test before using the cmRCT design to recruit participants into a cohort and then actual trials, since if the system was implemented as a means to improve trial recruitment and then proved unacceptable to participants it could jeopardise the trials.

### Research questions

In the DiReCT study we posed four questions:

1. The ‘Ethics Test’ – how ethically acceptable is the cmRCT?
2. The ‘Recruitment Test’ – what proportion of NHS patients with common mental health problems will agree to join a cmRCT cohort?
3. The ‘Trials Test’ – how many patients in the cohort would prospectively consent to being randomly selected to receive new treatments?
4. Clinical acceptability – what were the views of clinicians about the process of routinely recruiting patients into a cmRCT?

## Methods

### Study design

Our design was observational and uncontrolled in nature in that we did not conduct an experimental comparison of recruitment methods. We undertook a mixed methods study to test the feasibility of the cmRCT method for recruiting participants into a cohort which could then be used for RCT recruitment. In the first phase of the DiReCT study we tested the ethical acceptability of the cmRCT method; in the second phase we tested the recruitment yield; and in the third phase we investigated the views of participating clinicians.

### Setting and participants

We conducted the DiReCT study at a single clinical site in the south of the UK, part of a larger ‘Improving Access to Psychological Therapies’ (IAPT) service, treating people for common mental health problems, such as depression and anxiety. The study site receives over 3,500 patient referrals annually and covers a population radius of 62 km^2^. We recruited patients attending the service, aged 18 and older, during a 4-month period between November 2012 and February 2013. Patients were those being assessed and treated for common mental health problems by clinicians – including both junior Psychological Wellbeing Practitioners (PWPs) and senior therapists – who provided psychological treatment to patients. We also recruited a purposive sample of clinicians who had been involved in recruiting participants for the DiReCT study.

### Procedures

#### Phase I: the ethics test

In our first phase we applied for ethical permission to conduct the DiReCT study from the UK Health Research Authority (HRA), for review by a National Research Ethics Service Committee in order to determine if the HRA would find the features of the cmRCT methodology ethically acceptable, principally the ethics of seeking prior consent from participants for future randomisation into unspecified clinical trials. We developed our ethics application and participant materials with input from a member of a public and patient involvement (PPI) group for depression: the Lived Experience Group at the University of Exeter. We outlined procedures that sought three levels of consent from participants: (1) to be contacted by researchers; (2) to share their medical records with researchers; (3) to be offered randomly allocated active treatment procedures in future unspecified clinical trials. We applied for approval through the web based ‘IRAS’ system through which all UK clinical researchers must seek ethical clearance.

#### Phase II: the recruitment and trials test

In the second phase of the DiReCT study we tested the yield of the cmRCT design in terms of the numbers of participants with common mental health problems presenting for treatment who consented to: (1) be contacted by researchers; (2) share their medical records with researchers; (3) be offered randomly allocated active treatment procedures in future unspecified, and at this stage hypothetical, clinical trials. We emphasised that agreement to random selection would not infer consent to future trial interventions, but merely consent to be offered randomly selected active treatment procedures in future unspecified clinical trials. At this stage our proposal to participants would not include actual future trials. Potential participants were informed that the DiReCT study was hypothetical in nature as a feasibility proof-of-principle test of our pre-randomisation consent procedures. At the time of testing there were no trials running and participants were informed that they would not be offered trial participation at the time of the study or in the future as a consequence of their participation in this initial feasibility test.

We used a three-stage consent process. In stage 1, all new patients referred or self-referred to the study site were posted a patient information sheet and consent form with a compliments slip from the IAPT service introducing the DiReCT study. In stage 2, clinicians assessing and treating patients invited them verbally to participate during one of their first two clinical appointments. Patients who indicated that they wished to participate in the study signed a form consenting to one or more of the three levels of participation. Clinicians were instructed that they should seek to obtain consent from all patients, but that it was acceptable to delay information giving and the consent procedure for exceptionally distressed patients until a later appointment. We also collected blank consent forms for patients who declined to participate, signed and dated by clinicians so that we could measure the total number of patients who were offered the opportunity to take part in the DiReCT study by clinicians. No personal information was included on declined consent forms. In stage 3, a member of the research team contacted consenting patients by telephone within 14 days of the participant giving consent to verify their consent using the same three levels as stage 2. All eventual participants included in the study, therefore, gave informed consent at both stages 2 and 3 before any data was collected or recorded by the research team. We allocated all such consenting participants a unique ID number, recorded all data on a case report form and stored it securely in an anonymised database.

#### Phase III: clinical acceptability

After the phase II recruitment and trials test had been completed, we undertook interviews with a purposive sample of clinicians involved in the DiReCT study to investigate their views of recruiting and consenting participants. We sampled both PWPs and senior therapists, purposively selecting those who had recruited either small or large numbers of participants into the study. A post-doctoral researcher, independent of the DiReCT study team, unknown to the clinicians and experienced in qualitative interviewing, approached clinicians directly and carried out the interviews. All interviews were conducted on the telephone whilst the respondent was in a private space at his/her workplace. Interviews were planned to take between 15 and 30 minutes, and were audio recorded and transcribed verbatim. The accuracy of transcripts was checked by another member of the research team before analysis. Interviews were structured using an *a priori* defined topic guide to enquire about clinicians’ views of having been involved in the recruitment and consent procedure for the DiReCT study. All interviews started with one overarching question: “Recently you were part of a research study called ‘DiReCT’. During this study you were to invite patients to enrol in the study, and if they agreed to do so, to take their consent. Please tell us about your experiences of doing this”. Additional probing questions were used to help clinicians elaborate on their initial descriptions.

### Outcome measures

We recorded the total number of patients assessed and treated by the service during the data collection period. We recorded the number of consenting participants and their consent levels along with the number of blank consent forms signed by a therapist, indicating the number of patients who declined to participate. We collected routine demographic data on age, gender and employment status, and depression levels as measured by the Patient Health Questionnaire-9 (PHQ-9) [22] for consenting participants from the IAPT service routine clinical record database. We also obtained an anonymised report of the same demographic and clinical variables for the total patient population treated at the site during the study period.

### Analysis

We analysed the results of phase II using descriptive statistics to report: (1) the proportion of patients who were offered participation in the DiReCT study compared to the total number of patients assessed and treated at the service; (2) the proportion of patients consenting to participation at each level of consent compared to the number offered participation; and (3) the proportion of participants confirming consent to participate at each level of consent compared to the number initially consenting at the same levels. We calculated the similarities or differences between our sample and the total assessed and treated population on age, gender, employment status and PHQ-9 score using unpaired *t*-tests for continuous variables and chi-square for dichotomous variables.

Members of our team, experienced and trained in the analysis of qualitative data (DAR and SRos conducted a descriptive analysis of interview transcripts using a framework-guided thematic analysis [23]. We began analysis with familiarisation of the text and used our *a priori* topic guide to structure our initial descriptive coding scheme to develop categories of meaning. As concepts within these topics began to emerge, we used constant comparison techniques and pattern coding to connect emergent categories and refine our coding framework. An experienced and independent third member of the team (GB) oversaw and refined our analysis and advised on the further analysis and presentation of the qualitative data. We held regular meetings between analysts to establish the trustworthiness of the coding frame and thematic categories.

## Results

### Phase I: the ethics test

We obtained a favourable ethical opinion from the HRA’s National Research Ethics Service Committee East Midlands – Nottingham 1 Research Ethics Committee (REC) after receiving advice from the committee to submit for ‘Proportionate Review’. The Proportionate Review Service receives applications from research studies where the ethics committee believe that there is minimal risk, burden or intrusion for research participants and consequently applications are reviewed by a sub-committee rather than at a full meeting of a REC. The process of obtaining a favourable ethics opinion for the DiReCT study took 2 weeks from submission by this process (Ref 12/EM/0326). The sub-committee lead reviewer sought a telephone conversation with the Chief Investigator to clarify details of the application, during which there was an acknowledgement by the reviewer of the importance of improving trial recruitment, given that many ethics extension applications to RECs are a result of study recruitment difficulties. The reviewer raised no concerns about our intention to invite patients to pre-consent to randomisation as part of our hypothetical feasibility test, nor to the overall principle of our consent procedures should trials actually be running in any future cmRCT.

### Phase II: the recruitment and trials test

#### Consent data

The clinical service assessed 1,240 people during the 4 months of the DiReCT study, of whom 752 were offered treatment for depression and anxiety problems and were thus eligible for participation. All 752 treated patients were posted study information in advance of their first assessment. The clinical team obtained consent or decline data from 131/752 (17%) of potential participants during the study period. Of these 131 people, 47 (36%) declined to participate. Of the remaining 84 (64%), all participants consented to be contacted by a researcher, 83 consented to their medical records being looked at by researchers, and 83 consented to being randomised into future trials.

**Figure 1:**
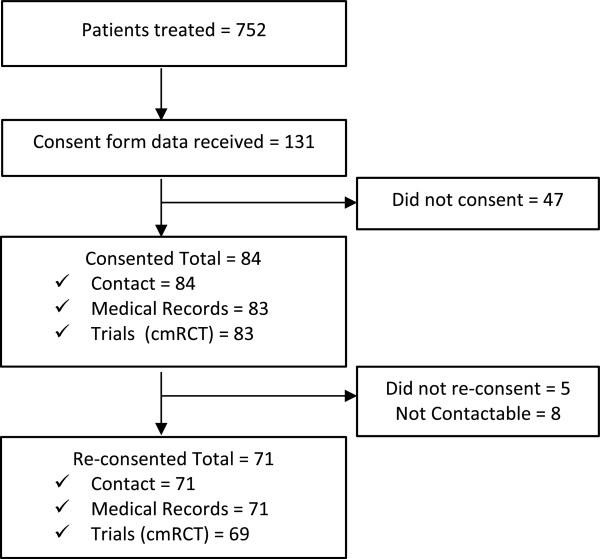
**Recruitment flow diagram.** cmRCT, cohort multiple randomised controlled trial.

When contacted by the research team to confirm consent, 71/84 (85%) of participants consented to having their medical records included in research and 69/84 (82%) consented to be offered randomly allocated active treatment procedures in future unspecified and at this stage hypothetical clinical trials. Five out of 84 (6%) people decided against participation and we were unable to contact the remaining eight (10%).Figure 1 summarises the flow of participant recruitment where 71/752 (9%) of the total number of patients who were offered treatment during the study period consented to take part and 69/131 (53%) of all patients who were invited by a clinician to take part consented to be offered randomly allocated active treatment procedures.

#### Participant demographics

There were no differences between included participants in age, gender, employment and depression severity (Table 1) when compared with the total number of people treated by the service during the study period, although there was a trend towards our included participants having a greater severity of depression.

**Table 1:** **Participant demographics**

	Study sample	Clinical population	***t***value or chi square	95% CI	***P***value
n	71	752			
Age (years; mean (SD))	41.5 (15.6)	42.1 (15.4)	0.31 (df =821)	−3.161 to 4.361	0.75
Gender	F =48 (67.6%); M =23 (32.4%)	F =452 (60.1%); M =300 (39.9%)	1.47 (df =1)	n/a	0.23
Employment status	Employed: 38 (53.5%)	Employed: 346 (46.0%)	1.42 (df =1)	n/a	0.24
PHQ9 score (mean (SD))	17.5 (5.3)	16 (6.3)	1.94 (df =821)	−3.017 to 0.017	0.053

df, Degrees of freedom; F, female; M, male; n/a, not applicable; PHQ9, Patient Health Questionnaire-9.

#### Consent procedure

Twenty-one clinicians were involved in the initial consent procedure: 14 senior therapists and seven PWPs. Sixteen clinicians were full time and five part-time. Table 2 details recruitment patterns by grade and hours worked.

**Table 2:** **Number of consent or decline forms returned by clinician type**

	n	Mean (SD)	***t***value	95% CI	***P***value
PWPs	7	12.9 (7.6)	3.36	4.21 to 15.99	0.013
Therapists	14	2.8 (3.3)			
Full time	16	6.9 (7.5)	0.92	−3.30 to 9.10	0.379
Part time	5	4 (5.7)			
All	21	6.2 (7.1)			

PWP, Psychological Wellbeing Practitioners.

### Qualitative interview data

We interviewed nine of the 21 clinicians who were involved in the DiReCT study (four therapists and five PWPs). All clinicians who were asked to take part in the interviews consented. Our analysis aimed to describe the views of clinicians about the process of routinely recruiting and consenting patients into the DiReCT study. Our results showed that their views could be understood in terms of four themes: ‘working with patients takes priority’; ‘workplace systems and pressures of daily work’; ‘clinicians’ views of the DiReCT project and of research; ‘clinicians’ perception of patients’ reaction to the recruitment process’.

#### Working with patients takes priority

The theme ‘working with patients takes priority’ describes the difficulties clinicians could experience in balancing their existing clinical practices with the recruitment process for the DiReCT study. The theme covered both their views on having to meet the patient’s needs in general, especially those patients in great distress, together with the importance clinicians ascribed to developing a therapeutic relationship.

In terms of meeting patients’ needs, clinicians described that they would only carry out the consent procedure provided their clinical priorities had been attended to first. Such priorities focussed on meeting patients’ immediate mental health needs. Introducing the DiReCT study was described as getting in the way of devising a treatment plan and delivering the therapy. Treating the patient's problems was viewed as a greater priority for clinicians than inviting them to take part in this study. They described having to make prioritisation decisions on when to introduce the topic of research participation - for example, at the second appointment when patients could be less anxious or even at a later appointment when clinicians were monitoring patient progress rather than introducing new clinical techniques.

“I was constantly trying to weigh up, you know, should I let this go or, what’s in the patient’s interest in this case? Should I pursue it or, let that go and focus more on the treatment and coming up with the rest of the plan?” (PWP 5)

“I think a lot of the time it was very, very difficult to do it… see, it was easier to perhaps broach it right at the beginning at the assessment, or, umm after you’ve had a few sessions because then, there was less going on”. (PWP 2)

In some cases clinicians described how they had to support patients who were in distress as the major focus of their sessional work for these patients. This was particularly the case when clinicians were interviewing patients in great distress, where the focus of their clinical work was very clearly directed towards responding to and helping the patient manage their distress. Introducing the study to distressed patients was described as making the clinicians feel uncomfortable. One clinician explained it as:

“I think… if someone is extremely distressed then… it’s not the first priority at that point when someone is in distress or upset… it wouldn’t feel comfortable at the end of an appointment that had been clearly very emotionally difficult for a patient to say, ‘Oh and by the way, can I just have a talk to you about this whole other thing”. (PWP 4)

Furthermore, in this theme the clinicians described the importance of the therapeutic relationship between themselves and patients. They expressed concern that introducing the DiReCT study would get in the way of developing this relationship, particularly at the early stages of their therapeutic work with patients.

“Having another element, right at the key point when you’re doing that relationship building with people… and you already feel like you’re not getting enough time to talk to them about what’s bringing them, and how they’re feeling at times…” (Therapist 1)

#### Workplace systems and pressures of daily work

The theme ‘workplace systems and pressures of daily work’ describes the experience of organisational prerogatives on clinicians’ daily work and how this affected their ability to recruit patients into the DiReCT study. This theme covered descriptions of workload targets and resulting time pressures, clinical roles in the team and administrative systems.

In regards to workload targets and resulting time pressures, clinicians described how high volumes of patients and short appointment times could make it difficult to fit the recruitment procedure into an appointment. One of the issues described in this theme was the clinicians’ concern over how to find the time they believed they needed in order to participate in the process of recruiting and consenting patients into the study. As one clinician expressed it:

“We’re so pressured with targets to get, you know, see so many people a week… and have 30 minute sessions, and then it’s sort of one after the other, you’ve barely got time between each session to even reflect. That adding anything else onto it… was very pressured.” (PWP 2)

Clinicians’ description of the recruitment process also included their views on the roles and responsibilities of different clinicians in the team. When it came to describing this, senior clinicians in a ‘high-intensity’ therapy role outlined how difficult it could be for them to recruit patients during the first few appointments, as they described being less familiar with this assessment role, ordinarily undertaken by PWPs. At times, because of staff shortages, senior therapists provided substitute cover for PWPs. In these circumstances, they described how not consistently seeing the same patients at follow up appointments could make it difficult to carry out the recruitment process if they had not been able to do so during the first appointment.

“Because we have an issue with the high attrition [of PWP’s], we’ve had a lot of [senior therapists] doing assessments, then put them on the waiting list for a PWP to pick up… so when the PWP picks up the person it might be a month between the assessment and the follow up, so they’ve got to… check the problem statement’s the same… and it’s a bit of a mini reassessment, so that takes time… they’ve also got to start some therapy, and then they’ve got to spend eight minutes going through the study… they were struggling to get all that in”. (Therapist 3)

Furthermore, within this theme of workplace systems and pressures of daily work, clinicians reported that the impact of different working conditions could in their view impede their ability to fit the recruitment and consent procedure into their appointments. For example, they described how a lack of equipment and record keeping systems could make it difficult to remember to invite patients to take part in the study.

“We had this, umm, additional new clinical space that they’d purchased for us upstairs, they didn’t have any IT and didn’t have any phones… so actually… what happened with that was it made it quite difficult… to err, remember when somebody was on their second or third or fourth session that was quite tricky…. and that meant that sometimes people were missed”. (PWP 5)

#### Clinicians’ views of the DiReCT project and of research

In the theme ‘clinicians’ views of the DiReCT project and of research’ the way clinicians viewed both research procedures specific to the DiReCT study and their perceptions of research in general were reflected.

In terms of clinicians’ experiences of the DiReCT project, they described how the complexity of the DiReCT recruitment and consent process and the amount of paperwork required could make it difficult for them to present the study to patients, and that having less of a role in the procedure would have been easier for them. It was felt by some clinicians that researchers should carry out informed consent and that clinicians should only have a limited role - for example, in collecting ‘consent to contact researcher’ forms from patients. A less involved role might have persuaded clinicians to approach more patients. The hypothetical and pilot nature of the DiReCT study was also viewed as a reason why clinicians might choose not to try and recruit some patients, given that recruiting patients would confer no tangible benefits to their patients.

“I’d have been quite happy for someone to ask people, you know, to bring me something and if they bring it to me, just tick say, ‘I’m interested in more information on this, but I’m not signing up to anything right now’… I’d be quite happy for that and then let the research team talk about it with them or something because they know what’s going on better than I do… so it’s just a little bit more streamlined so we have less of a role in terms of explaining”. (PWP 1)

“As far as I was aware it was almost like a pre-test to, pre-research so it wasn’t actually kind of… as far as I was aware it wasn’t actually counted towards anything so again it was a bit confusing”. (Therapist 4)

Clinicians described generally positive attitudes to research and how having experience of research in general could contribute to a positive attitude to the DiReCT study. Again, this could reflect the way that clinicians viewed their role in research, in this case a more general attitude to research *per se*. Having a background in research was described as being useful in helping clinicians feel more competent in carrying out the procedure, which some clinicians viewed as getting easier with practice. A positive aspect of the DiReCT study was that it was viewed as possibly helping to contribute to future quality research, which was described as important.

“There were some things which I had liked about it because I do think it is important that we get umm… you know, umm… lots of people to make really robust, umm… research papers written umm but, so that was the good bit, that was me, sort of quite liking that aspect of it”. (PWP 1)

#### ‘Clinicians’ perception of patients’ reaction to the recruitment process’

The theme *‘*clinicians’ perception of patients’ reaction to the recruitment process’ contained descriptions around how patients reacted when the clinicians approached them in regards to the recruitment and consent process. The theme also covered ethical concerns around the validity of consent consequent upon interpersonal interactions reported by clinicians.

Clinicians described how it could be more difficult to recruit certain patients. They were less likely to invite patients to participate in the DiReCT study if he or she displayed characteristics, views or behaviours that were viewed as not conducive to the recruitment and consent procedure. One clinician described it as:

“We have a bit of an issue with err, angry men… so, umm who, we’ll have some people who come in quite angry and they don’t want to fill the MDS in so, if they’re saying ‘I really-, I’m not filling this in’, err and they’re quite confrontational… then we’re not going say ‘well can you fill-, can I spend eight minutes to discuss this with you as well?’” (Therapist 3)

In this theme the clinicians additionally described that there was a distinct divide in attitude between those patients who were willing to consent and those who were not willing. Clinicians described how they were more likely to follow through the recruitment process with patients who showed an apparently positive attitude to the study already. For example, some patients would attend appointments having made their decision to participate in the DiReCT study prior to attending their assessment, in that they brought the consent form already signed to their first appointment.

“Yeah the people, that was quite distinctly the people that, umm, did want to do it, it was quite obvious, the people that did… their view was ‘Ooh yes, we need something like this, I’ve read it through, and’ …oh yeah, I mean the people that wanted to do it had read it all the way through, the people that didn’t want to do it, a few of them had read it through and didn’t see the point, and a few of the others had been put off by it and sort of thought ‘I don’t wanna do this”. (PWP 3)

Patient characteristics as perceived by clinicians also affected their decisions over whether to offer participation - for example, low levels of patient literacy which could lead clinicians to doubt patient comprehension. Clinicians also reported their perception that those patients with more severe depression were less likely to consent to participation. Some clinicians described feeling confused about the eligibility of newly referred patients who wanted to take part but were not eligible for treatment by the service.

“It was quite tricky when… if they’d received it in the post and they had got enthusiastic about it but then I’d deemed them not suitable, because they weren’t suitable for our service, that was a bit difficult”. (PWP 5)

The ethical concerns perceived by the clinicians were mainly described as reflecting the validity of consent consequent upon interpersonal interactions between clinicians and patients. Descriptions covered how the clinicians questioned the validity of consent from patients who did not appear to fully understand the DiReCT study. It could become apparent for the clinicians that the patients had signed the consent form without fully reading the patient information sheet. One clinician described it as: “I mean ‘fully understood’ is pushing it in some cases, I mean some of them told me they hadn’t read it, they’d just signed it” (PWP 1)*.* These kinds of situations were described as making the clinicians feel uncomfortable about forwarding their consent forms to the research team.

“I had a couple of people that… had come in, signed it all and I just kind of checked it out and they said ‘Oh I just signed it, I don’t really care, you know I just want to get on with this’… so I wasn’t quite sure what to do with that really that they’d signed it, they said ‘yes I’m consenting’, but… when I’d come to explain the bit about the study, they really weren’t interested”. (Therapist 3)

Further, the theme also represented descriptions concerning the interpersonal relationship dynamic between the clinician and patient, where the clinicians perceived that they were unintentionally endorsing the DiReCT study to the patients. Clinicians also described how the power dynamic within the therapeutic relationship could make discussing the study with patients make them feel uncomfortable.

“I was also concerned as well about the kind of the, power aspect of the relationship as well you know, I was awfully aware that, you know, with the best will in the world, we always try not to be in a position of power, that’s part of the role but, you are… umm and asking someone to go away and read this thing, explaining… why it’s important or why it’s being done… you’re kind of, endorsing it, you’re kind of saying this is something that’s good, ‘it’s your choice’ but ‘it’s good’… and it just, it feels a little uncomfortable”. (PWP 1)

## Discussion

We found that more than 50% of patients in a routine UK NHS anxiety and depression service would consent to become part of a cohort within which they could be randomly selected to receive future unspecified treatments in clinical trials, a research design that was acceptable to a UK ethics committee. The acceptability of the cmRCT system to clinicians was influenced by their clinical concerns for patient wellbeing, pressures of work, their views of both general research and the specific DiReCT study, and how they viewed patients’ responses to being offered participation in the study.

In the UK at least, ethics committees are prepared to agree to a system where only those people randomised to active trial arms are subsequently approached by researchers, and where those in the control group are not informed about their trial participation. We have replicated the result of a previous favourable ethical opinion [20] that the cmRCT – a form of modified Zelen design – is an ethically acceptable alternative to recruitment into the standard RCT design where all participants are approached for specific trial consent. In our application of the cmRCT principles, participants were pre-warned about the possibility of random selection and asked to consent, albeit to unspecified experimental treatments, before being included in the cohort. Our DiReCT study recruitment and consent procedures may, therefore, address some ethical reservations about the original Zelen design.

Our results for our recruitment and trials tests, regarding the willingness of patients in a routine health care setting to agree to both inclusion in a cohort and to be randomly selected for trial treatments, are very encouraging. All but two of our recruited participants were willing to be enrolled in both the cohort itself and random selection. As a consequence, we might predict that if it was standard practice to offer all patients suffering from common mental health disorders the opportunity to participate in future unspecified clinical trials, around 50% of patients would consent to participate in both the cohort and future trials. By consenting to both cohort membership and trial participation, patients were agreeing to provide data as part of a potential control group for trials, without further consent being required. In previous trials we have found that, when identifying and approaching potential participants directly after screening patient records, only around 10% of potentially eligible people are subsequently randomised [24]. Thus, the cmRCT may present a method of maximising participant recruitment that could be around five times more efficient than alternatives. One might also argue that trials that recruit 50% of potentially eligible participants are likely to produce results that are more generalisable to the clinical population under study.

Less encouraging was the fact that clinicians held recruitment and consent discussions with only 17% of potentially eligible participants. Indeed, other commentators [9, 14] have noted that a major barrier to participant recruitment is clinician, not patient, reluctance to engage in the research recruitment process. The results from our analysis of clinician interviews help to explain our descriptive statistical analyses. Our clinicians described a range of factors that could influence their decision-making, including their prioritisation of patient care, the practical difficulties of finding time to engage with patients about research, their own views about the research process itself and how patients responded when asked to participate. Some clinicians experienced a conflict between their research recruitment responsibilities and the need to establish a therapeutic relationship and care for distressed patients, a finding echoing previous studies [11, 25, 26]. Overlaying these interpersonal concerns were organisational difficulties including the pressures of work, wherein clinicians had multiple clinical and administrative jobs to do within the context of a highly pressured environment, once again an issue described in other studies [11]. The hypothetical nature of the DiReCT study was cited as a reason not to engage in recruitment as was the need to offer complex research design explanations.

### Strengths and limitations

Our DiReCT study was hypothetical in nature. We did not have any trials running in a cmRCT system at the time we undertook this feasibility study. Therefore, we are unable to report the conversion rate of those participants who agreed to be part of the pre-randomisation consenting cohort and those who would subsequently actually consent to the offer of an experimental intervention. It is conceivable that some of our DiReCT cohort might turn down the offer of treatment once it was made concrete. Conversely, our yield might have been higher if trials had been running, given the reticence of some clinicians to offer patients the opportunity to participate in what was only a hypothetical exercise in this instance, and the unknown number of patients who might have declined participation for the same reason.

Our data is reliant on clinician self-report. We had no way of verifying if the numbers of blank consent forms returned represented the true number of participants who were offered and then declined participation. It might be that we have over-estimated the proportion of participants who would consent to be part of a cmRCT system once they are asked by a clinician, since it might be that some participants were asked but that clinicians did not return a blank form. However, a 50% participation rate is not unusual in previously reported studies [27, 28] lending credence to our observations.

Our study was small, particularly in terms of the numbers of clinicians interviewed, a limitation on the findings from our interview analysis. Although we would urge caution in accepting our interview data as generalisable to other clinical teams, there have been similar views reported in several other studies [9, 25]. It is certainly possible that our analytical themes will be relevant beyond our specific contextual environment, but replication elsewhere should be undertaken. Furthermore, we did not collect qualitative interview data from participants, which may have given us further insights into the acceptability of the cmRCT design from the perspective of potential patient participants.

### Implications

The implications of the DiReCT study are two-fold. Firstly, despite the fact that our study was hypothetical and that there was no direct benefit to participants, more than 50% of patients approached consented to participation. One might regard our results, therefore, as a conservative estimate of the potential cmRCT yield if trials were actually recruiting. We also suggest that the diagnoses of patients might not have a serious impact on the system yield, given that in UK cancer services 64% of patients who were approached to take part in research subsequently went on to do so [29], only slightly more than in our community mental health environment. As noted by the originators of the cmRCT model [3], the cmRCT system could provide a ready-made pool of research participants already having consented to the offer of trial treatments. Trialist and health care providers would then avoid the need to set up new recruitment procedures each time a trial was initiated in the same health care environment.

Secondly, it is clear that engaging clinicians in the research process is both difficult and yet as equally important as the not inconsiderable efforts being made to improve PPI in research, particularly in the UK, where extensive PPI is a prerequisite for NIHR project funding. Clinicians gave us multiple, sometimes contradictory, explanations for their recruitment behaviours. For example, one finding from our qualitative data was that clinicians did not have a consistent view on how much they should be responsible for collecting informed consent from their patients. Some clinicians suggested confining themselves to merely recruiting patients for contact with researchers, and that researchers should explain the nature of the research being proposed, effectively shifting responsibility for study recruitment from clinicians to researchers. Indeed, such a system of ‘consent for consent’ (for example, http://www.slam.nhs.uk/research/patient-involvement/current-opportunities/consent-for-contact) is a feature of some research active health care providers’ activities. However, other clinicians wanted to assure themselves that patients truly understood what they were consenting to, before being willing to co-sign consent forms. In contrast, pressure of work and clinical focus were more universal explanations offered by clinicians to explain their difficulties recruiting participants.

Clearly, health care providers could consider embedding a cmRCT system in their routine clinical procedures as one method to address the findings of a recent report that suggested many health care providers are not orientated towards research participant recruitment [30]. However, multiple efforts are likely to be required to engage many clinicians. One possibility suggested by Donovan and colleagues [11] is for more support and training of clinicians. However, this may be insufficient in of itself, and a more comprehensive implementation model might be required, including attention to individual clinician, organisational and informational factors. One such model, the Normalisation Process Theory [31], proposes that in order to embed new procedures in routine practice efforts have to be made to enable all concerned to make sense of the new system (‘coherence’), build and sustain a community of practice around it (‘cognitive participation’), undertake the operational work to enact a set of new practices (‘collective action’), and appraise the work that people do to assess and understand the ways that a new set of practices affect them and others around them (‘reflexive monitoring’). It is likely that such theoretically underpinned and sophisticated implementation procedures will be required before the recruitment of participants into research cohorts becomes embedded in routine practice, given that the research community has been reporting these difficulties for many, many years [25].

## Conclusion

The cmRCT system offers the potential to improve the efficiency of recruitment into clinical trials and is acceptable ethically and to many patients. Nonetheless, overcoming the multiple factors driving the difficulties clinicians experience in patient recruitment more generally is likely to require the application of significant implementation science-informed effort.

## Authors’ information

David A Richards, PhD, BSc (Hons), RN is Professor of Mental Health Services Research and Sarah Ross BSc (Hons) is Associate Research Fellow at the University of Exeter Medical School, UK. Sarah Robens, PhD, MA, BSc (Hons) is a Research Fellow at Devon Partnership NHS Trust, Exeter, UK, a partner clinical service provider of the University of Exeter. Gunilla Borglin PhD, MSc, RN is a Senior Lecturer at Malmo University, Sweden.

## Abbreviations

cmRCT: cohort multiple randomised controlled trial
HRA: Health Research Authority
IAPT: Improving Access to Psychological Therapies
MDS: minimum data set
NHS: National Health Service
NIHR: National Institute for Health Research
PHQ-9: Patient Health Questionnaire-9
PPI: public and patient involvement
PWP: Psychological Wellbeing Practitioner
RCT: randomised controlled trial
REC: Research Ethics Committee.
